# EQUINOXE study: Impact of relational cohesion and sexuality on the quality of life of patients treated with gonadotropin‐releasing hormone agonist for prostate cancer

**DOI:** 10.1002/bco2.92

**Published:** 2021-10-19

**Authors:** Stéphane Droupy, Marie‐Hélène Colson, Nathalie Pello‐Leprince‐Ringuet, Valérie Perrot, Aurélien Descazeaud

**Affiliations:** ^1^ Department of Urology & Andrology CHU Caremeau Nîmes France; ^2^ Department of Immunology Hematology CISIH, Ste Marguerite Hospital Marseille France; ^3^ Ipsen Boulogne‐Billancourt France; ^4^ Department of Urology University Hospital Limoges France

**Keywords:** dyadic adjustment, gonadotropin‐releasing hormone, prostate cancer, quality of life, relational cohesion

## Abstract

**Objectives:**

To measure the effect of dyadic adjustment on changes in patients’ quality of life when initiating treatment with gonadotropin‐releasing hormone (GnRH) agonist.

**Patients and methods:**

A prospective, multicenter, longitudinal, and non‐interventional study (NCT02630641) that included patients with prostate cancer starting GnRH agonist therapy, and their partners, in 157 centers in France. Data were collected at inclusion and after 6 months of treatment on quality of life (WHOQOL‐BREF), disease perception (B‐IPQ), disease symptoms (QLQ‐PR25), and perception of cohesion within the couple (dyadic adjustment, DAS‐16).

**Results:**

The Full Analysis Set included 492 patients (median age [Q1;Q3]: 74 [68;80] years). An improvement of the quality of life (defined as the improvement of at least one of the four dimensions of WHOQOL‐BREF) was reported in 290/434 (67%) patients between baseline and follow‐up. Quality of life was better at baseline and follow‐up in patients with good cohesion within the couple than in those with medium or poor cohesion. Factors associated with improvement in quality of life of patients were the following: initial presence of QLQ‐PR25 hormonal treatment‐related symptoms (OR [95% CI]: 3.00 [1.46, 6.17]) suggesting testosterone deficiency symptoms at baseline and initial low level (2.04 [1.12, 3.72]) or absence of sexual activity (2.23 [1.11, 4.50]) before GnRH agonist initiation.

**Conclusion:**

Men with the greatest improvement in quality of life after initiating hormone therapy were those with, at baseline, testosterone deficiency symptoms (identified by QLQ‐PR25 treatment‐related symptoms score) or no/low sexual activity. Cohesion within the couple was not confirmed as an influence on the evolution of quality of life.

## 1 INTRODUCTION

Prostate cancer is the most common form of cancer affecting men in France. It is estimated that over 50,430 cases were diagnosed in 2015 in France and that prostate cancer is the third cause of male cancer‐related deaths. In 2018, prostate cancer was believed to have been responsible for 8,115 deaths in France.^1^ Consequently, prostate cancer places a significant burden on public health, and improvements in the quality of treatment and quality of life of prostate cancer patients constitute an important public health need.

Treatments available for more advanced prostate cancer include hormone therapy with gonadotropin‐releasing hormone (GnRH) agonists, including goserelin, leuprorelin and triptorelin, (±anti‐androgens), in combination with radiation therapy or as monotherapy, depending on the tumor stage.

A diagnosis of prostate cancer, or a relapse of previously treated prostate cancer, remains a traumatic experience for the patient.^2^, ^3^, ^4^ There is a sudden disruption to the patient's everyday life, leading to anxiety and uncertainty about the future. Furthermore, because prostate cancer requires prolonged treatment, this can impose considerable constraints on the patient.^2^, ^4^, ^5^, ^6^. The patient's social environment and, in particular, support from the patient's partner and the positive impact of a satisfactory marital relationship may have a major effect on their quality of life.^7^, ^8^, ^9^, ^10^ The degree of cohesion in the relationship may have a major effect on acceptance of the disease and its treatment, and in modifying the quality of life both of patients and their partners.^3^, ^8^, ^9^, ^11^, ^12^, ^13^

Previous research on the effects of social environment and relationship cohesion in prostate cancer has been limited to isolated evaluation of the prostate cancer patient or his partner, but seldom performed on the couple both at the initiation of treatment and during follow‐up, and on the interactions between the two parts of the dyad: patient and partner. This observational study, involving a national sample of urologists in France, aimed to obtain information on the effect of the cohesion of the couple, namely the dyadic adjustment, on the quality of life of patients with prostate cancer initiating GnRH agonist therapy.

## 2 PATIENTS AND METHODS

### 2.1 Ethics

The study was conducted in compliance with the Declaration of Helsinki, 2008, the International Ethical Guidelines for Epidemiological Studies, CIOMS, Feb.2008, the International Epidemiological Association Guidelines for Proper Conduct in Epidemiologic Research (GEP) 2007, and International Society for Pharmacoepidemiology (ISPE) Good Pharmacoepidemiological Practices (GPP) Guidelines 2007.

Before starting the study, the protocol, patient information sheet and all other documents were submitted to the relevant authorities, in compliance with the French legislation. Written informed consent was obtained before enrollment and data collection.

### 2.2 Study design

This prospective, multicenter, observational, longitudinal, non‐interventional study was conducted at 157 centers in France from October 2015 to November 2017 (ClinTrials.gov Number NCT02630641). Patients initiating GnRH agonist treatment and their partners were given self‐questionnaires at the enrollment/baseline visit and at the follow‐up visit 6 months later. The decision to prescribe a GnRH agonist was made in accordance with local clinical practice prior to and independently from the decision to enrol the patient.

### 2.3 Physician recruitment

Practicing urologists in France, who had been identified as prescribers of GnRH agonists for the treatment of prostate cancer, were recruited from a cohort of 1627 urologists from a database maintained by CEGEDIM (Boulogne‐Billancourt, France), an independent company. Invitations to participate were sent to this cohort and the first 250 to return a written agreement were recruited.

### 2.4 Patients

#### 2.4.1 Inclusion criteria

Patients were eligible for participation in the study if they met the following criteria: histologically confirmed prostate cancer; eligible to start GnRH agonist therapy, either as monotherapy or adjuvant therapy, and for whom one of these treatments was selected voluntary by the urologist before the start of the study; and living with current partner for at least 6 months. The patient and partner had to be capable of reading, understanding, and completing and returning a self‐questionnaire at baseline and 6 months after the initial consultation.

#### 2.4.2 Exclusion criteria

Patients were excluded if they were participating in another clinical trial or had received GnRH therapy within the previous 2 years.

### 2.5 Outcome assessments

The study included two visits: an enrollment visit (baseline), which was the patient's most recent medical assessment, at which the decision to initiate GnRH agonist treatment was made; and a follow‐up visit as close as possible to 6 months after the enrollment visit. At each visit, patients and their partners were given their corresponding blank questionnaires. The patients and partners were asked to return the completed questionnaires (which they completed separately) by post in the envelopes provided within 1 month of the enrollment visit and after the 6‐month follow‐up visit; late questionnaires (>45 days after visit) were excluded.

#### 2.5.1 Effectiveness assessments

Patients and partners were asked to complete three self‐questionnaires at enrollment (the standardized French World Health Organization Quality‐of‐Life Scale [WHOQOL‐BREF], the Dyadic Adjustment Scale [DAS‐16], and the Brief Illness Perception Questionnaire [B‐IPQ]) and two at the follow‐up visit (WHOQOL‐BREF and DAS‐16), and patients completed an additional quality of life scale specific for prostate cancer (QLQ‐PR25) at enrollment and follow‐up.

The WHOQOL‐BREF includes 26 items exploring two single items—the individual's overall perception of quality of life (first item) and the individual's overall perception of their health (second item)—and four dimensions (physical health, psychological health, social relationships and environment), each comprising three to eight items. Each item is answered on a five‐point scale.^14^, ^15^ The scores of each single item and each domain were transformed to a 0‐100 scale, a higher score denoting a better quality of life. For an individual, a change of at least 1 point in item 1 or item 2 scores in the 0‐100 scale is clinically meaningful, it means a change from one to another class in the five‐point scale.

The DAS‐16 includes 16 items allowing the evaluation of a general dyadic adjustment factor and two sub‐dimensions, which are the degree of agreement in the couple (DAC) and the quality of marital interactions (QMI).^16^, ^17^ Each item was scored on a six‐point scale, and the DAS‐16 total score was transformed to a 0‐154 scale (DAS). The higher the score, the higher the person's perception of having a good dyadic adjustment. Clinical thresholds for classifying patients were DAS <92 = poor adjustment; 92≤ DAS ≤107 = medium adjustment; DAS >107 = good adjustment. A couple has an inconsistent level of dyadic adjustment if one person has a DAS >107 and the other has a DAS <92.

The B‐IPQ was completed only at baseline. It comprises eight items assessing cognitive illness representations, emotional representations, and illness comprehensibility.^18^ Each of the eight items is scored on a scale from 0 to 10, and the total score varies from 0 to 80. A higher score reflects a more threatening view of illness.

The QLQ‐PR25, completed by patients at both visits, comprises 25 items grouped into five multi‐item scales, and one single item: urinary symptoms (eight items); bowel symptoms (four items); hormonal treatment‐related symptoms (six items); sexual activity (two items); sexual functioning (conditional on being sexually active; four items); and the single item (bother due to the use of incontinence aid).^19^ The five subscores and the single item were transformed to a 0‐100 scale. Higher scores represent more symptoms/problems.

#### 2.5.2 Safety assessments

As this was a non‐interventional study, adverse event (AE) reporting was to follow regulations related to spontaneous cases, ie, investigators were asked to report related AEs and serious AEs (related or not) to the safety department of the drug manufacturer using the usual process for such reactions. Safety data listings were obtained from the Ipsen safety database and from case report forms.

### 2.6 Statistical analysis

The sample size was calculated based on the hypothesis that 20% of patients would experience an improvement in the quality of life dimension during the follow‐up period. We assumed that 15 patients in each explanatory parameter [eg dyadic adjustment, QLQ‐PR25 treatment‐related symptoms, …] would experience an improvement in the quality of life, with a minimum of 10 explanatory parameters. Therefore 150 patients presenting with an improvement in the quality of life would be required, ie a total of 750 patients to reach the hypothesis of 20% of patients with an improvement would be required. To take account of incomplete data and patients ineligible for assessment, a target of 1,000 enrolled patients was planned. Statistical analysis was performed using Statistical Analysis System (SAS^®^) version 9.3 (SAS Institute Inc, Cary, NC, USA). Three populations were defined in this study: the recruited population, all patients for whom at least one study document was collected; enrolled population, all patients who gave informed consent after the initiation of the study and before the date of end of inclusion; and the Full Analysis Set (FAS) comprising all enrolled patients with at least one baseline and follow‐up (at 6 months) patient's self‐questionnaire, and no major protocol deviation. All analyses were performed in the FAS. All the statistical tests were performed for exploratory purposes only.

#### 2.6.1 Primary endpoint

The primary effectiveness endpoint was the change in the quality of life of the patient measured by WHOQOL‐BREF from baseline to follow‐up in the FAS. An improvement of the quality of life was defined as the improvement of at least one of the four dimensions of WHOQOL‐BREF (physical health, psychological health, social relationships, and environment), and multivariable logistic regression modeling was applied to this endpoint. The variation from baseline for the two single items and each dimension were presented quantitatively, and the significance of the change was evaluated with a paired Student's *t*‐test.

#### 2.6.2 Secondary endpoints

Secondary endpoints included change in DAS, change in QLQ‐PR25, the effects of baseline factors including the dyadic adjustment on improvement in patient's and partner's quality of life, and change in quality of life of the partner (WHOQOL‐BREF).

The total scores of the DAS were presented at baseline and follow‐up visit for the patient and the partner. Score variations were presented, and the significance of the change was evaluated with a Student's *t*‐test.

The effects of demographic and clinical parameters as well as patient's DAS, B‐IPQ, and QLQ‐PR25 scores at baseline on change in patient's quality of life were identified using a logistic regression model. Univariable logistic regression analysis was used for each baseline parameter separately. Factors were selected for multivariable analysis if statistically significant at level *α* ≤ 0.20, and independence between factors was studied. Multivariable stepwise logistic regression model involving all factors selected in previous step, including their interaction, if relevant (significant at *P* <.10), was performed. The significance level was set at 5%.

The first single item of the WHOQOL‐BREF was employed as the main evaluation of partner quality of life evolution. An improvement of partner quality of life was defined as the improvement of the first single item, and logistic regression model was applied to this endpoint. The variation from baseline for the two single items and each dimension were presented quantitatively, and the significance of the change was evaluated with a paired Student's *t*‐test.

## 3 RESULTS

### 3.1 Study population

**FIGURE 1:**
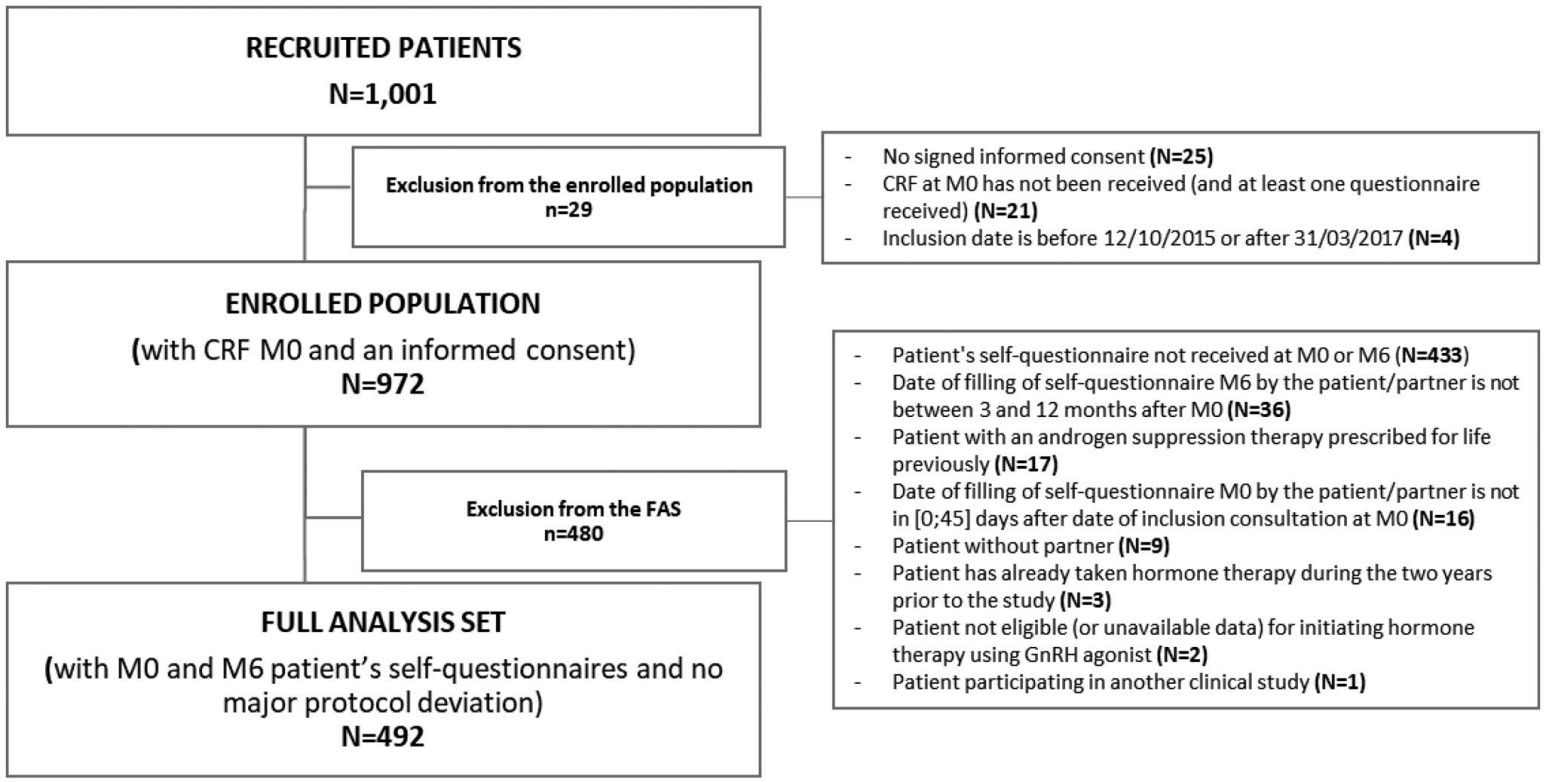
Patient disposition. M0, month 0 (baseline); M6, Month 6; CRF, Case report form; GnRH, Gonadotropin‐releasing hormone

A total of 1,001 men were recruited between October 2015 and March 2017; and 972 (97%) gave informed consent and were enrolled, 752 of whom (77%) completed the study. The FAS comprised 492 patients (Figure 1), and results presented for partners include only the partners of these 492 men. Baseline data for patients in the FAS are presented in Table 1. Baseline demographics, medical, and oncological characteristics of patients in the FAS were similar to baseline data of patients not included in the FAS (data not shown). Median [Q1;Q3] ages of patients and partners, respectively, were 74 [68;80] (n = 492) and 71 [64;77] years (n = 470); median [Q1;Q3] length of relationship was 40 [26;50] years (n = 450); except 2, all the partners were women (n = 460).

**TABLE 1:** Baseline characteristics of patients—Full Analysis Set (n = 492)

Characteristics	n	Results
Patient age (years), median [Q1;Q3]	492	74 [68;80]
*Patient's level of physical activity, n (%)*		
Active (sports, walks >30 minutes day)	491	279 (57)
Sedentary (no sport, does not walk >30 minutes day)	491	212 (43)
*Patient comorbidities n (%)*		
Any	492	325 (66)
Hypertension	325	211 (65)
Ischemic cardiopathy	325	49 (15)
Dyslipidemia	325	106 (33)
Diabetes	325	90 (28)
Osteoporosis	325	10 (3.1)
Neuropsychological disorders	325	20 (6.2)
Other	325	57 (18)
*Prostate cancer diagnosis history, n (%)*		
New diagnosis	491	356 (73)
Relapse^a^	491	135 (27)
*TNM classification at time of diagnosis, n (%)*		
T < 3, N0/NX, M0/MX	481	159 (33)
T ≥ 3, N0/NX, M0/MX	481	182 (38)
All T, N1, M0/MX	481	48 (10)
All T, all N, M1	481	92 (19)
*Symptoms, n (%)* ^b^		
Urinary symptoms	492	200 (41)
Sexual symptoms	492	203 (41)
Other symptoms (asthenia, anorexia, bone pain)	492	121 (25)
*Hormonal treatment, n (%)*		
GnRH agonist alone	488	330 (68)
Complete androgen blockade	488	158 (32)
*Objective of GnRH agonist treatment, n (%)*		
Salvage therapy after local treatment	489	95 (19)
Neoadjuvant to radiotherapy	489	198 (40)
Palliative care for a locally advanced or metastatic stage	489	196 (40)
*QLQ‐PR25 score (/100), mean (SD)*		
Urinary symptoms	487	23.1 (19.0)
Problem related with an incontinence aid^c^	104	36.9 (33.1)
Bowel symptoms	482	10.8 (17.0)
Hormonal treatment‐related symptoms	487	16.2 (15.8)
Sexual activity	485	69.5 (25.7)
Sexual functioning	328	46.6 (25.2)

Note

Abbreviations: GnRH, Gonadotropin‐releasing hormone; SD, standard deviation.

^a^ Prior treatments in patients who relapsed: radiotherapy (53%); prostatectomy (53%).

^b^ One patient can have more than one symptom.

^c^ Only in patient with an incontinence aid.

Most men (73%) were receiving GnRH agonist treatment (triptorelin, leuprorelin, or goserelin) in the context of a new diagnosis of prostate cancer; 66% had comorbidities (Table 1).

At baseline, partners had a worse perception of the patients’ illness than the patients themselves (mean [SD] B‐IPQ total score (/80): 44.0 [7.2] (n = 385) and 39.8 [9.2] (n = 463), respectively).

### 3.2 Quality of life

An improvement in at least one of the four dimensions of patient WHOQOL‐BREF between baseline and the 6‐month follow‐up visit (primary endpoint) was reported by 290/434 (67%) patients.

**FIGURE 2:**
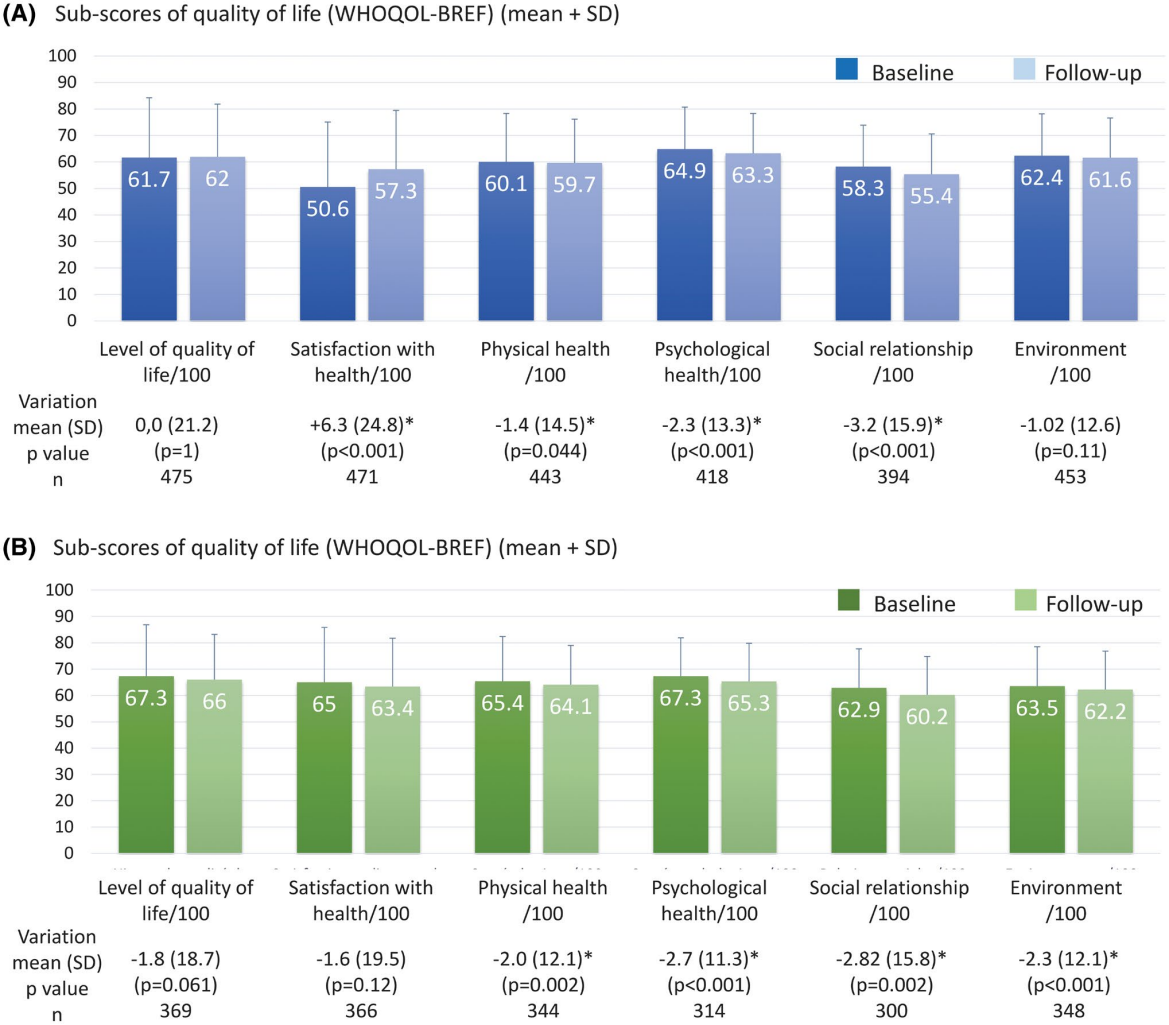
Quality of life WHOQOL‐BREF score for (A) patients and (B) partners—Full Analysis Set. * Significant evolution (*P* <.05); p‐value paired Student's *t* test. Bars are mean +SD, SD, standard deviation; WHOQOL‐BREF, World Health Organization Quality‐of‐Life Scale; range 0‐100, higher scores mean better evaluation

In the 6 months after starting GnRH agonist therapy, patients’ quality of life (first item of WHOQOL‐BREF) remained mostly stable, while patients’ satisfaction with their health (second item) improved significantly (mean change was +6.3 points/100 and clinically meaningful). Physical and psychological health, and social relationship scores worsened significantly (mean changes were −1.4 to −3.2 points/100) during the 6‐month follow‐up (Figure 2A).

An improvement of the partner's quality of life (first item of WHOQOL‐BREF) between baseline and the 6‐month follow‐up visit was reported by 56/369 (15%) of partners. Partners’ mean scores for quality of life (first item) and satisfaction with their health (second item) remained stable. Physical health, psychological health, social relationship, and environment scores worsened significantly (mean changes were −2.0 to −2.8 points/100) over the 6‐month follow‐up (Figure 2B).

### 3.3 Dyadic adjustment and impact on quality of life

**FIGURE 3:**
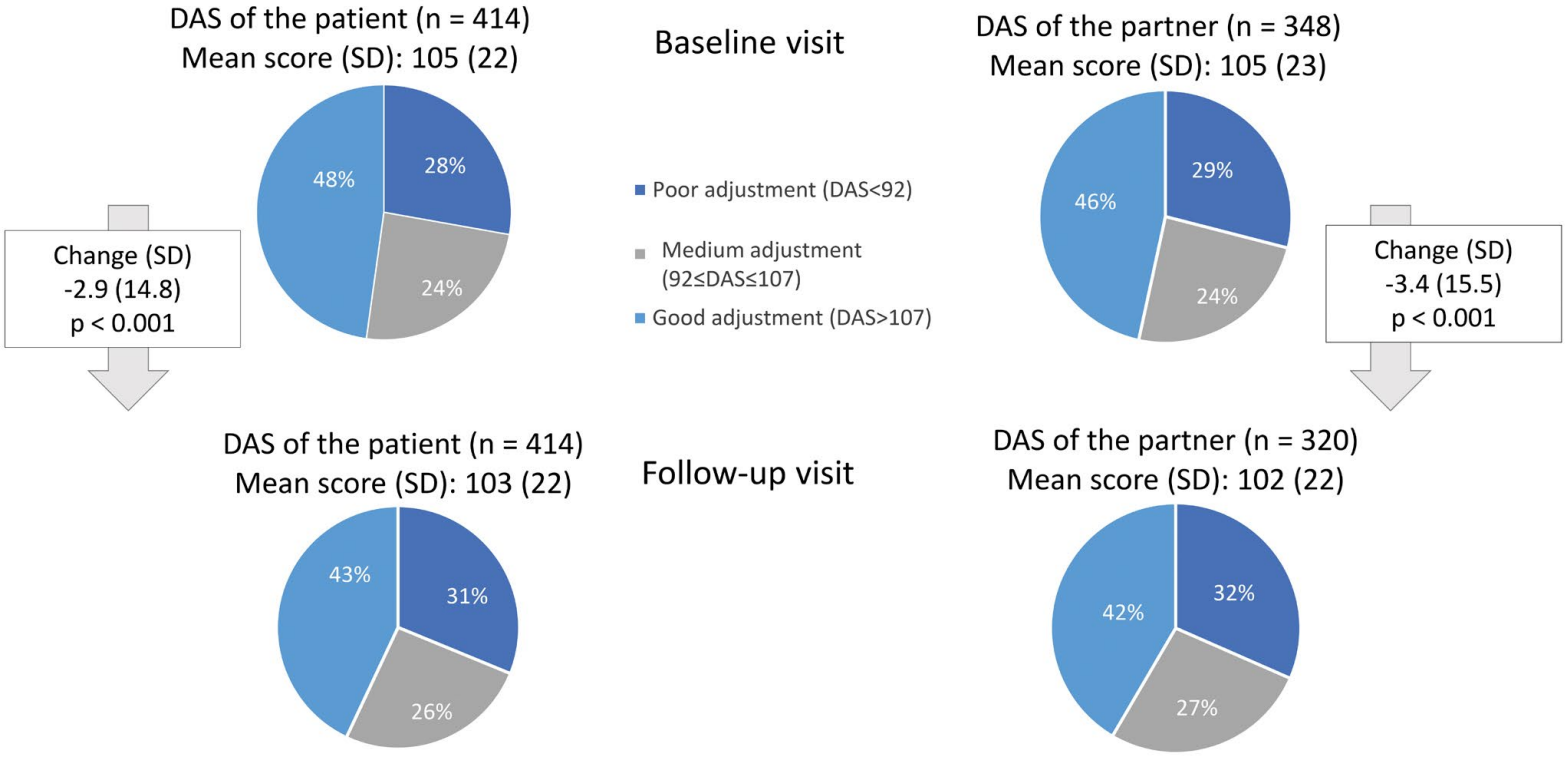
DAS of the patient and partner—Full Analysis Set. DAS, Dyadic Adjustment Scale; range 0‐154, higher scores mean better dyadic adjustment; p‐value paired. Student's *t*‐test

A high proportion of both patients (48%) and partners (47%) reported a good dyadic adjustment at baseline (Figure 3). Over the 6 months of follow‐up, the couples’ cohesion deteriorated, with the mean DAS total scores decreasing significantly (Figure 3). DAS scores of patient and partner were in similar ranges—for 96% at baseline and for 96% at the 6‐month follow‐up visit—showing low levels of inconsistency within couples.

Both at baseline and follow‐up, patients’ quality of life measured by the first single item in the WHOQOL‐BREF was numerically higher in patients who had good cohesion with the couple (DAS) than in patients with medium or poor cohesion (Table 2). The same was observed with the other five dimensions (data not shown). Quality of life (first single item) evolution over the 6‐month follow‐up in patients who had good, medium, and poor cohesion with the couple at baseline was significantly different (*P* = .014) (Table 3). Overall good cohesion with the couple seemed to be an unfavorable factor for the evolution of the quality of life score, evaluated by the first item.

**TABLE 2:** WHOQOL‐BREF questionnaire scores, at baseline, and at follow‐up, for patients, for the single item, “How would you rate your quality of life? (/100)” according to dyadic adjustment group (DAS score), respectively at baseline and at follow‐up—Full Analysis Set (n = 492)

Baseline first item score according to baseline adjustment (DAS)	N	Mean (SD)	Median [Q1;Q3]
Poor adjustment	113	52.0 (24.1)	50 [25;75]
Medium adjustment	101	61.4 (21.4)	75 [50;75]
Good adjustment	194	68.8 (19.7)	75 [50;75]

Follow‐up first item score according to follow‐up adjustment (DAS)	N	Mean (SD)	Median [Q1;Q3]
Poor adjustment	128	52.9 (21.0)	50 [50;75]
Medium adjustment	105	60.7 (19.3)	75 [50;75]
Good adjustment	177	68.9 (17.1)	75 [50;75]

Note

DAS, dyadic adjustment scale; DAS <92 = poor adjustment; 92≤ DAS ≤107 = medium adjustment; DAS >107 = good adjustment; SD, standard deviation; WHOQOL‐BREF, World Health Organization Quality‐of‐Life Scale.

**TABLE 3:** Change of WHOQOL‐BREF questionnaire scores, from baseline to follow‐up, for patients, for the single item, “How would you rate your quality of life? (/100)” according to dyadic adjustment group (DAS score) at baseline—Full Analysis Set (n = 492)

Evolution of first item score according to baseline adjustment (DAS)	n	Mean (SD)	Median [Q1;Q3]	P‐value
Poor adjustment	110	1.6 (22.5)	0 [0;25]	
Medium adjustment	99	2.8 (19.8)	0 [0;0]	.014
Good adjustment	194	‐4.1 (19.8)	0 [−25;0]	

Note

DAS, dyadic adjustment scale; DAS <92 = poor adjustment; 92≤ DAS ≤107 = medium adjustment; DAS >107 = good adjustment; SD, standard deviation; WHOQOL‐BREF, World Health Organization Quality‐of‐Life Scale; p‐value Kruskall–Wallis test.

Patient factors associated with the improvement in patient's quality of life in univariable analyses (*P*‐value < .20) were DAS of the patient, urinary symptoms (QLQ‐PR25), hormonal treatment‐related symptoms (QLQ‐PR25), sexual activity (QLQ‐PR25), age, context of cancer management, level of physical activity, B‐IPQ total score of patient, duration of the relationship, hormone therapy (neoadjuvant to radiotherapy versus salvage therapy after local treatment versus palliative care for a locally advanced metastatic stage), having at least one sexual function disorder, and having at least one other clinical symptom.

In the multivariable analysis, factors associated with the improvement in the patient's quality of life were initial presence of QLQ‐PR25 hormonal treatment‐related symptoms, and initial low level or absence of sexual activity (Table 4). For partners, factors associated with an improvement of quality of life were low cohesion of the couple, initial presence of QLQ‐PR25 hormonal treatment‐related symptoms in the patient, and initial absence of sexual functioning by the patient (Table 4).

**TABLE 4:** Multivariable analysis: patients’ factors associated with an improvement in patients’ quality of life improvement (defined as improvement of at least one out of four dimensions of WHOQOL‐BREF) and patients and partners factors associated with partners’ quality of life improvement (defined as improvement of WHOQOL‐BREF first item score)—Full Analysis Set (n = 492)

Patients multivariable analysis (n = 331)	Reference	OR (95% CI)	*P*‐value
*QLQ‐PR25: treatment‐related symptoms*			
Between 0 and 25/100	0/100 (no symptom)	1.68 (0.95‐2.97)	
≥25/100	0/100 (no symptom)	3.00 (1.46‐6.17)	.012
*QLQ‐PR25: sexual activity*			
Between 50 and 100/100	<50/100	2.04 (1.12‐3.72)	.04
100/100 (no activity)	<50/100	2.23 (1.11‐4.50)	

Partners multivariable analysis (n = 217)	Reference	OR (95% CI)	*P*‐value
*Dyadic adjustment of the partner (DAS total score)*			
Poor adjustment	Good adjustment	9.61 (3.18‐29.01)	<.001
Medium adjustment	Good adjustment	1.68 (0.46‐6.16)	
*QLQ‐PR25: treatment‐related symptoms*			
≥25/100	<25/100 (no or few symptoms)	5.99 (2.40‐14.93)	<.001
*QLQ‐PR25: sexual functioning*			
Between 50 and 100/100	<50/100	0.64 (0.25‐1.60)	
100/100 (no functioning)	<50/100	14.95 (2.49‐89.92)	.004

Abbreviations: CI, confidence interval; OR, odds ratio.

### 3.4 Safety

Twelve patients reported a total of 30 AEs during the study: five deaths not otherwise specified, 24 serious AEs reported by six patients (five of whom died: two from general health deterioration, one from septic shock following peritonitis, one from hemorrhagic stroke after hypertensive crisis, one end‑stage disease progression), and one related non‐serious AE (hot flushes) in one patient. Ten patients died during the study; according to the investigators, none of the deaths was related to the study drug.

## 4 DISCUSSION

Prostate cancer patients’ perception of their illness, and their relationship with their partner, is of great importance in the patient's and partner's ability to cope with the illness and in improving their quality of life. This prospective, multicenter, longitudinal, non‐interventional study, conducted in France, investigated the evolution of quality of life in patients with prostate cancer, and their partners, following initiation of GnRH agonist therapy, and examined the importance of relational cohesion. Characteristics of the patient population were similar to those of two previous large observational studies in France, conducted in populations initiating, respectively, a 1‐year (n = 1,438) or a 2‐year (n = 891) GnRH agonist treatment, both mainly involving urologists like our study.^20^, ^21^ For example, median [Q1;Q3] age of patients at baseline was 74 [68;80] years in our study, and the mean (SD) age of patients was 74.9 ± 8.1 and 74.1 ± 8.7 years in the other studies, respectively; the percentage of patients with any comorbidities was 66% in our study vs 65% and 72%; 19% of our patients had metastatic prostate cancer vs 23% and 21%; and locally advanced stages (T3‐T4 or N1) accounted for 48% of our patients vs 44% and 52%, indicating that our sample was representative of this patient population in France.

In our study, patients’ quality of life (evaluated by the first item of WHOQOL‐BREF) remained stable over the 6 months following initiation of GnRH agonist therapy. However, 67% of the patients reported an improvement of the quality of life in one of the four domains (primary endpoint), and the initial presence of hormonal treatment‐related symptoms and low level or absence of sexual activity were associated with this improvement. This may be caused by these factors resulting in a lower quality of life at baseline, and hence these men may have had less potential for quality of life to deteriorate after initiation of hormone treatment (and even a greater potential for improvement). Although the greatest deterioration in quality of life was observed for patients with good cohesion with the couple, those patients’ quality of life at baseline and follow‐up remained higher than quality of life of patients who had medium or poor cohesion with the couple. The multivariable analysis did not confirm the influence of the cohesion within the couple on quality of life for the patient. However, it underlined the importance of testosterone deficiency symptoms (as measured by QLQ‐PR25 hormonal treatment‐related symptoms score) and of lack of sexual activity at baseline.

The quality of life of partners of patients treated for 6 months with GnRH agonists for prostate cancer tended to deteriorate. However, three parameters were associated with an improvement of partners’ quality of life: low cohesion of the couple, initial presence of testosterone deficiency symptoms in the patient, and initial absence of sexual functioning in the patient. These results highlight the necessity of future investigations on partner's quality of life. In the couple perspective, it seems that the partner's quality of life followed the same evolution as the patient's quality of life, and that individuals belonging to a couple with poor intimacy before initiation of hormonal treatment may both see their quality of life raise during patient's hormonal treatment. This improvement may be because there is low potential for intimacy to worsen, and patient and partner thus perceive only the benefit of hormonal treatment.

Limitations of our study include its observational design and the high rate of patients not included in the FAS (Figure 1). The main reason for exclusion was that the patient's M0 or M6 self‐questionnaire was not received (433/480 [85.1%]); according to the protocol and inclusion criteria, patients were responsible for returning their questionnaires, which included data for the primary endpoint (patient's WHOQOL‐BREF). Nevertheless, the rate of improvement in the WHOQOL‐BREF was higher than expected (67% instead of 20%); thus, fewer patients than anticipated were needed to support the analysis. Because the purpose of this study was mainly oriented toward the couple, collection of data that can impact patients’ quality of life was not exhaustive. For example, data on types of radiotherapy administered in the neoadjuvant and salvage stages and concomitant systemic treatments prescribed in the advanced stages were not collected; consequently, their potential impact on quality of life could not be evaluated. Concerning the choice of the endpoint and the minimum clinically meaningful change on WHOWOL‐BREF scores, no cut‐off was described previously in the literature. The choice of an increase in the score to consider an improvement in any of the domains was therefore arbitrary and even +1 point was considered an improvement. Sensitivity analyses were performed using different cut‐offs of +1 to +10 points, the agreement between the primary outcome using the different cut‐offs was high.

Strengths of our study include its prospective design and the collection of data, in real life, on patients and their partners, during the first 6 months of initiation of GnRH agonist treatment. To our knowledge, this is the largest cohort of prostate cancer patients providing data from the partners of the patient. Few studies have provided data from both patients and partners in prostate cancer or other cancers, while even fewer provide dyadic data. A prospective evaluation of 191 metastatic breast cancer patients and their partners to assess the effect of dyadic coping on the well‐being of the patient and their partner highlighted the important role of the couple in managing the stress associated with the illness.^22^ Likewise, a study in 31 men treated for oral cancer and their partners indicated that overall quality of life was higher in patients and partners living in stable relationships.^23^ A study in 149 patients with recent cancer diagnoses and their partners highlighted the importance of parallel examination of both members of the couple to achieve better adaptation to the illness.^24^ A pilot study has been conducted in 28 couples to assess the pre‐operative barriers using DAS to cope with the sexual side effects after radical prostatectomy for prostate cancer.^25^ A systematic meta‐analysis of studies conducted in breast and prostate cancer populations reported that couple‐based interventions had small but beneficial effects in terms of improving multiple aspects of quality of life, including psychological and relationship‐based outcomes for both patients and their partners.^26^

## 5 CONCLUSION

The findings of this study give important new information on the couple cohesion assessed by DAS questionnaire in a wide population of patients with prostate cancer initiating GnRH agonist therapy. They also bring new data on the relation between couple cohesion and patients’ quality of life: the higher the couple cohesion is, the higher the quality of life of patients is, both at baseline and after a 6‐month GnRH agonist therapy which deteriorated several dimensions of their quality of life. The patients who benefited the most from hormone therapy in terms of quality of life were those who suffered from testosterone deficiency symptoms and those with no/low sexual activity at baseline. This would indicate the need for a more comprehensive psycho‐sexual support in patients without these characteristics at baseline. Our results highlight the importance of assessing the couple relationship at baseline as a way of offering support options adapted to the degree of change expected.

## DISCLOSURES

Stéphane Droupy: Ipsen, Menarini, Majorelle, AMS, Intuitive Surgical, Sanofi, Pierre Fabre, Takeda, Ferring. Marie‐Hélène Colson: Allergan, Astellas, Astra‐Zeneca, Bayer santé familiale, Biopharm, Boston, Bouchara‐Recordati, Ferring SA, Genévrier, Ipsen, Lilly SA, Majorelle, Menarini, Novartis, Pfizer santé de la famille. Aurélien Descazeaud: Bouchara Recordati, Ipsen, Sanofi, Pierre Fabre, Takeda. Nathalie Pello‐Leprince‐Ringuet and Valérie Perrot are employees of Ipsen.

## Data Availability

Where patient data can be anonymized, Ipsen will share all individual participant data that underlie the results reported in this article with qualified researchers who provide a valid research question. Study documents, such as the study protocol and clinical study report, are not always available. Proposals should be submitted to DataSharing@Ipsen.com and will be assessed by a scientific review board. Data are available beginning 6 months and ending 5 years after publication; after this time, only raw data may be available.
